# Physicochemical dataset from Lima and Douro estuaries (Northwest Portugal)

**DOI:** 10.1016/j.dib.2025.111317

**Published:** 2025-01-21

**Authors:** Vânia Freitas, C. Marisa R. Almeida, Sabrina M. Rodrigues, Rúben Pereira, Ana M. Gorito, Jacinto Cunha, Diogo M. Silva, Alexandre van Heerden, Sandra Ramos

**Affiliations:** a CIIMAR/CIMAR LA, Interdisciplinary Centre of Marine and Environmental Research, University of Porto, Terminal de Cruzeiros do Porto de Leixões, 4450-208 Matosinhos, Portugal; b Chemistry and Biochemistry Department, Faculty of Sciences, University of Porto, Rua do Campo Alegre, 687, 4169-007 Porto, Portugal; c Institute of Biomedical Sciences Abel Salazar (ICBAS), University of Porto, Rua de Jorge Viterbo Ferreira n° 228, 4050-313 Porto, Portugal; d Biology Department, Faculty of Sciences, University of Porto, Rua do Campo Alegre, s/n, 4169-007 Porto, Portugal; e CITAB/Inov4Agro – Centre for Research and Technology of Agro-Environmental and Biological Sciences, University of Trás-os-Montes and Alto Douro, Vila Real, Portugal

**Keywords:** Water quality, Nutrients, Metal levels, Monitoring, Transitional waters

## Abstract

This article presents a comprehensive dataset of physicochemical data from two urban estuaries on the northern Portuguese coast, based on five sampling campaigns conducted between October 2021 and November 2022. The dataset includes: a) *in-situ* vertical profiles of water physicochemical parameters (temperature, salinity, pH, turbidity, dissolved oxygen concentration and saturation) collected at 8 sampling stations along the Lima estuary (during both ebb and flood tides) and at 11 stations along the Douro estuary (during flood tides); and b) chemical analyses data, including nutrients (nitrate, nitrite, phosphate, ammonium, and silica), chlorophyll *a*, total particulate matter, particulate organic matter, and key metal (copper, zinc, cadmium, iron, nickel, lead, manganese, and chromium) concentrations. For the Lima estuary, additional data on dissolved metals concentrations are provided, offering a detailed picture of metal contamination. This dataset provides valuable insights into the estuarine dynamics of two important temperate systems, with a particular focus on anthropogenic influences such as nutrient enrichment and metal contamination. The data have strong reuse potential in environmental monitoring, providing a baseline for assessing anthropogenic changes and offering quality-assured references for studies linking contaminant distribution with hydrodynamic patterns in estuarine environments. Furthermore, such data are relevant to supporting the implementing of environmental policies, such as the EU Water Framework Directive for transitional waters.

Specifications Table

**Table:** 

Subject	Environmental Science
Specific subject area	Environmental chemistry and Estuarine water quality
Type of data	Table, Figure Raw, Analysed, Filtered.
Data collection	Vertical profiles of physicochemical water parameters (temperature, salinity, pH, dissolved oxygen saturation, dissolved oxygen concentration, and turbidity) were measured using a YSI EXO1 Sonde multiparameter probe. Surface and near-bottom water samples were collected with a Hydro-Bios 2 L sampler to analyse nutrients (NO_3_^−^, NO_2_^−^, PO_4_^3−^, NH_4_^+^, Si), particulate matter, and metals (Cu, Zn, Cd, Fe, Ni, Pb, Mn, Cr), in the laboratory. Nutrients and chlorophyll *a* concentrations were measured with a VWR® V-1200 Visible Spectrophotometer. Metal concentrations were determined using atomic absorption spectrophotometry (PerkinElmer AAnalyst 200 or PinAAcle 900Z with AS 900 autosampler).
Data source location	Two estuaries located in the NW Portugal: Lima (41°40′ N and 8°50′ W) and Douro (41°08′ N and 8°40′ W) (Fig. 1). The geographical coordinates of each sampling station are indicated in Table 1, Table 2.
Data accessibility	Repository name: Zenodo Data identification number: 10.5281/zenodo.13868241 Direct URL to data: 10.5281/zenodo.13868241
Related research article	None

## 1 Value of the Data

• These datasets provide baseline concentrations of nutrients and metals, which are essential for long-term environmental monitoring and for detecting pollution events or ecosystem changes in these two urban estuaries and adjacent coastal waters. This information also facilitates comparisons with other estuarine systems.
• It provides *in-situ* measurements that capture short-term (tidal) and long-term (seasonal) temporal variations, enhancing the understanding of estuarine dynamics throughout the year. This knowledge is also critical for accurate water quality assessment and the design of effective monitoring programs in estuaries with similar hydrodynamic conditions.
• Data on metal concentrations provide essential insights into the chemical status of these two transitional systems, supporting compliance with European environmental directives such as the Water Framework Directive (WFD) and the Marine Strategy Framework Directive (MSFD).
• Data from multiple sampling stations ensure a comprehensive spatial analysis of physiochemical parameters and contaminants useful for studies linking contaminant distribution to estuarine hydrodynamic patterns, and can assist in the identification of potential sources of contamination.
• These quality-assured datasets, generated using standardized methods, are hosted in an open repository to support further scientific research, validation and practical application in environmental policy and management. Emerging applications include the integration of these data into international/national datasets (e.g., EMODnet) in support of Blue Digital initiatives, and into hydrodynamic and biogeochemical models to predict the response of estuarine water quality to changing environmental conditions.

## 2 Background

This dataset was compiled to provide a detailed understanding of the physicochemical parameters and contaminant dynamics (nutrient and metal concentrations) in the Lima and Douro estuaries. In addition to establishing baseline concentrations of nutrients and metals, it provides accurate *in situ* measurements that can help to elucidate their behaviour throughout the year, including interactions with natural temporal and tidal variations. Furthermore, the dataset also aims to identify potential contamination sources within the estuarine environment. This information is important to support effective monitoring and the implementation of regional management plans aimed at maintaining and improving water quality and the estuarine ecosystem health.

## 3 Data Description

**Fig. 1:**
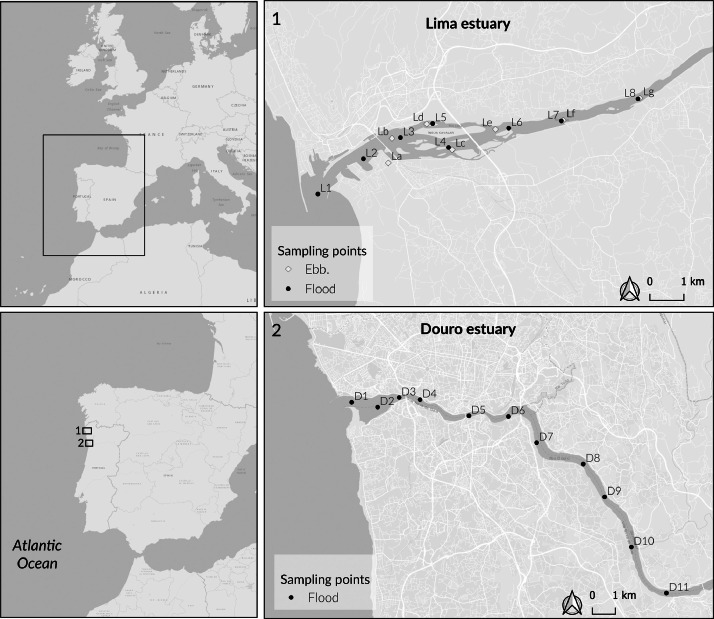
Location map showing the two study areas: Lima (1) and Douro (2) estuaries, in northwest Portugal. The geographical coordinates of the sampling stations are given in Tables 1 and 2.

The dataset described in this data paper contains physicochemical water data from five sampling campaigns conducted at two temperate North Atlantic estuaries, the Lima and Douro, between 2021 and 2022 (Fig. 1). The dataset is stored in Zenodo data repository and is accessible as an open file for download [1]. There are two separate Excel files, LimaEstuary.xlsx and DouroEstuary.xlsx, one for each estuary, each with two data sheets. The first sheet, “Vertical profiles”, contains general sampling information (e.g., sampling date, tidal phase, station name, latitude and longitude in decimal degrees (WGS84, EPSG:4326)) and the *in-situ* profiles of temperature, salinity, pH, turbidity, dissolved oxygen saturation percentage, and dissolved oxygen concentration, acquired with a multiparameter YSI EX01 probe. These parameters were measured for several sampling stations in Lima estuary (7 stations sampled during ebb tide and 8 stations sampled during flood tide) (Table 1), and in Douro estuary (11 stations sampled during flood tide) (Table 2), along the horizontal salinity gradient. The second sheet, “Chemical parameters”, contains data from laboratory chemical analyses of surface and near-bottom water samples for 5 stations in each estuary, considered representative of the different estuarine reaches. It includes two sets of measurements: i) nutrients, chlorophyll *a*, total particulate matter, and particulate organic matter concentrations; and ii) metal concentrations in particulate matter. In the Lima dataset (LimaEstuary.xlsx), measurements of dissolved metals concentrations in water are also included as a result of a more detailed study on metal concentrations conducted during one of the sampling campaigns (February 2022). For each chemical parameter, the mean and standard deviation of triplicate measurements (for nutrients) or duplicate measurements (for metals) are reported. Missing values due to sensor malfunction, equipment failure, measurement errors, and/or sample loss are indicated using specific notations. Values below the detection limit (LOD) are also indicated, along with their respective LODs.

**Table 1:** Geographical coordinates of sampling stations (decimal degrees, WGS84, EPSG:4326) in the Lima estuary.

Tidal phase	Sampling Station	Latitude	Longitude
Ebb	La	41.6872	−8.8200
	Lb	41.6937	−8.8144
	Lc	41.6907	−8.7931
	Ld	41.6975	−8.8021
	Le	41.6961	−8.7778
	Lf	41.6985	−8.7538
	Lg	41.7043	−8.7267
Flood	L1	41.6790	−8.8406
	L2	41.6883	−8.8245
	L3	41.6939	−8.8114
	L4	41.6913	−8.7944
	L5	41.6976	−8.8000
	L6	41.6964	−8.7731
	L7	41.6983	−8.7545
	L8	41.7041	−8.7274

**Table 2:** Geographical coordinates of sampling stations (decimal degrees, WGS84, EPSG:4326) in the Douro estuary.

Tidal phase	Sampling Station	Latitude	Longitude
Flood	D1	41.1449	−8.6683
	D2	41.1431	−8.6549
	D3	41.1468	−8.6437
	D4	41.1460	−8.6330
	D5	41.1397	−8.6077
	D6	41.1394	−8.5873
	D7	41.1292	−8.5727
	D8	41.1209	−8.5486
	D9	41.1081	−8.5376
	D10	41.0886	−8.5238
	D11	41.0707	−8.5057

## 4 Experimental Design, Materials and Methods

### 4.1 Study area

The Lima and Douro estuaries are located on the northwest coast of Portugal (Fig. 1). The Lima estuary is at the end of an international watershed covering 2522 km^2^, of which half are located in Portuguese territory. The estuary had a mesotidal regime (3.7 m) with semidiurnal tides extending up to 20 km upstream, and an average annual freshwater flow of 70 m^3^s^−1^ [2]. Situated near the city of Viana do Castelo, it represents an urban-industrialized environment subject to multiple disturbances [2,3]. The lower estuary is influenced by shipping and port activities, while the entire estuary is exposed to diffuse contamination from agriculture, industrial operations, and urban development. Agricultural runoff, domestic wastewater, and industrial effluents contribute to the discharge of nutrients and a range of substances, including metals and organic contaminants, into the estuary [4,5]. Additionally, ongoing dredging of the navigational channel in the lower estuary poses the risk of resuspending contaminants from the sediment into the water column [3]. Despite significant modifications, the estuary still contains intertidal saltmarsh areas and natural banks, which serve as important habitats for several ecologically and economically relevant fish species [6] and provide several ecosystem services [7], namely contributing to good surface water quality by acting as important pollutant filters.

The Douro estuary, located 65 km south of the Lima, is part of the river system with the largest watershed on the Iberian Peninsula. This highly urbanized estuary lies between Porto, Gondomar and Vila Nova de Gaia, three densely populated urban centres, and is significantly impacted by urban, industrial and agricultural activities [8,9]. With a semidiurnal and mesotidal regime, the estuary is artificially limited by a hydropower dam situated 22 km upstream, which strongly regulates freshwater inflow and the estuarine hydrodynamics [10]. The Douro is susceptible to anthropogenic contamination, receiving discharges from a total of eight wastewater treatment plants (WWTPs), with two of the largest facilities located along its lower and middle reaches [8,11]. Many studies have already documented contamination throughout the entire estuary ranging from microbiological [2] to chemical contamination, including metals [12,13].

### 4.2 Sampling and data collection

Sampling in the Lima and Douro estuaries was conducted between October/November 2021 and November 2022, in a total of five sampling campaigns, following methodologies described in [2]. In the Lima estuary, sampling was performed during spring tides of both ebb and flood tides, whenever possible on the same day, while in the Douro estuary, sampling was performed only during spring flood tides. Sampling took place during the day and in days with no precipitation events, starting 2 h before high-water slack for flood tides and 2 h before low-water slack for ebb tides. Sampling stations were distributed along the horizontal gradient of both estuaries, with coordinates provided in Table 1, Table 2. In the Lima estuary, 7 stations were sampled during the ebb tide and 8 stations during the flood tide, while in the Douro estuary, 11 stations were sampled. The number of sampling stations was proportional to the size of each estuary and was chosen to cover the salinity gradient of each estuary, according to the limits of the transitional waters defined for the Portuguese implementation of the WFD [14]. At each sampling station, physicochemical parameters (temperature, salinity, pH, dissolved oxygen concentration and percent oxygen saturation, and turbidity) were measured throughout the water column using a multiparameter probe (YSI EXO1 Multiparameter Sonde), each sensor properly calibrated according to the manufacturer's instructions with standard solutions. Additionally, water samples (surface and near-bottom) were collected from a subset of representative stations to assess nutrient levels, including nitrate ion (NO_3_^−^), nitrite ion (NO_2_^−^), phosphate ion (PO_4_^3−^), ammonium ion (NH_4_^+^), and silica (Si) concentrations, as well as total particulate matter (TPM), particulate organic matter (POM), chlorophyll *a*, and metal concentrations. Metals analysed included copper (Cu), zinc (Zn), cadmium (Cd), iron (Fe), nickel (Ni), lead (Pb), manganese (Mn) and chromium (Cr), which are commonly found along the northwest Portuguese coast [e.g., 15]. Metals have been measured mainly in particulate matter, with dissolved metals assessed during one sampling campaign (February 2021) in the Lima estuary.

Surface water (0–0.5 m depth) was collected directly filling 1.5 L plastic bottles, while near-bottom water was collected using a standard water sampler (Hydro-Bios 2 L) and then transferred into 1.5 L plastic bottles. All bottles were pre-decontaminated by immersion in a 10–20 % nitric acid (HNO_3_) solution for 24 h, rinsed with deionised water, and further washed *in situ* before sampling. Water samples were transported to the laboratory in a cool box and processed immediately upon arrival to the laboratory.

### 4.3 Analytical procedures

In the laboratory, water samples were filtered through 0.45 µm cellulose membrane filters (cellulose acetate for chlorophyll *a,* and cellulose nitrate for nutrients and metals analyses) and pre-combusted GF/F glass-fibre filters (for TPM and POM levels). Nutrients and dissolved metal levels (when measured) were determined from filtered water samples, with each sample analysed in triplicate for nutrients and in duplicate for metals. Chlorophyll *a* (filtration of 500 mL of water per sample), metals in particulate matter (each sample in duplicate, filtration of 250 mL of water per replicate), TPM and POM (filtration of 500 mL of water per sample) were determined using their respective filters.

All reagents used were pro analysis grade or equivalent. To prevent cross-contamination, all lab materials used were washed with deionised water (conductivity < 0.1 μS cm^−1^), immersed in a HNO_3_ solution (10–20 % v/v) for 24 h, washed again with deionised water and dried in a clean oven at 30 °C.

Nutrients (Si, NO_2_^−^, PO_4_^3−^, and NH_4_^+^) concentrations were quantified spectrophotometrically using a VWR® V-1200 Visible Spectrophotometer, following the methods described in [16]. Nitrate was quantified using an adaptation of the spongy cadmium reduction technique [17], with NO_2_^−^subtracted from the total. A calibration curve obtained with aqueous standard solutions of different nutrient ion concentrations (0–2 µM for NO_2_^−^, 0–5 µM for PO_4_^3−^, 0–20 µM for NH_4_^+^, 0–40 µM for NO_3_^−^) was used for each nutrient ion quantification. Blank solutions were always prepared and blank signal considered in the calibration curve. The detection limits were 0.02 µM for nitrite ions, 0.05 µM for phosphate ions, 0.1 µM for ammonium ions, 0.2 µM for silica, and 0.5 µM for nitrate ions. Chlorophyll *a* concentrations were measured by spectrophotometry (VWR® V-1200 Visible Spectrophotometer) after extraction of the filter with 90 % acetone [18], with cell homogenization following the SCOR-UNESCO trichromatic equation [19]. TPM was quantified following overnight drying of the GF/F glass-fiber filters at 100 °C, while POM was quantified by combustion at 500 °C, with filters weighed before and after [20]. Blank filters were always subject to the same procedure and their mass subtracted.

Metal (Cu, Zn, Cd, Fe, Ni, Pb, Mn, Cr) concentrations were determined by atomic absorption spectroscopy (AAS) following procedures validated before in the laboratory [3,15]. For dissolved metals, water samples were preconcentrated using a metal-chelating resin (Chelex 100) and eluted with HNO_3_, followed by AAS analysis (SpectrAA 220 FS, Varian) using flame atomization (FA) (Marck 7, Varian) or electrothermal atomization (ET) (Autosampler GTA 110, Varian), depending on metal levels [15]. A blank sample (deionised water) was subjected to the same procedure and metal signals subtracted from those of samples. For metals in particulate matter, filters were previously digested with concentrated HNO_3_ in a high-pressure microwave system (ETHOS 1, Milestone) (5 ml per filter) before AAS analysis which was carried out in a FA AAnalyst 200 and ET PinAAcle 900Z coupled to an AS 900 furnace autosampler (PerkinElmer), depending on metal levels. Blank filters were also digested and metal signals subtracted from those of samples. A calibration curve obtained with aqueous standard solutions of different metal concentrations (0–3 mg/L) was used for each metal quantification [3,15]. These standard solutions were prepared in deionised water (acidified with 1 % HNO_3_) from 1000 mg/L stock standard solutions of each metal. Blank solutions were always prepared and blank signal considered in the calibration curve. For quality control, doped samples were prepared and analysed along with samples, with recovery values always between 80 and 120 %. The detection limits for dissolved metals were 0.1 µg/L for Cu, Pb and Mn, 0.4 µg/L Zn, 0.01 µg/L for Cd, 2 µg/L for Ni and 0.02 µg/L for Cr. For metals in particulate matter, detection limits were 1 µg/L for Cu, Pb, Zn, Fe and Mn, 0.5 µg/L for Cr and Ni and 0.01 µg/L for Cd.

## Limitations

In Lima estuary, no sampling was conducted during flood tide in September 2022. Additionally, due to technical constraints, no water samples were collected during the November 2021 flood tide sampling campaign. As a result, data on nutrients, chlorophyll *a*, total particulate matter (TPM), particulate organic matter (POM) and metal concentrations are unavailable for that period.

## Ethics Statement

The authors have read and follow the ethical requirements for publication in Data in Brief and confirming that the current work does not involve human subjects, animal experiments, or any data collected from social media platforms.

## CRediT authorship contribution statement

**Vânia Freitas:** Investigation, Supervision, Data curation, Writing – original draft. **C. Marisa R. Almeida:** Conceptualization, Funding acquisition, Investigation, Methodology, Supervision, Writing – original draft, Data curation. **Sabrina M. Rodrigues:** Investigation, Data curation, Writing – review & editing. **Rúben Pereira:** Investigation, Data curation, Writing – review & editing. **Ana M. Gorito:** Investigation, Writing – review & editing. **Jacinto Cunha:** Investigation, Visualization, Writing – review & editing. **Diogo M. Silva:** Investigation, Data curation. **Alexandre van Heerden:** Investigation, Data curation. **Sandra Ramos:** Conceptualization, Funding acquisition, Investigation, Data curation, Supervision, Writing – review & editing.

## Data Availability

ZenodoA dataset of physicochemical water parameters from Lima and Douro estuaries (Northwest Portugal) (Original data)

## References

[1] Freitas V., Almeida C.M.R., Rodrigues S.M., Pereira R., Gorito A.M., Cunha J., Silva D.M., van Heerden A., Ramos S. (2024). A dataset of physicochemical water parameters from Lima and Douro estuaries (Northwest Portugal) (v1.0.0) [Data set]. Zenodo.

[2] Ramos S., Cabral H., Elliot M. (2015). Do fish larvae have advantages over adults and other components for assessing estuarine ecological quality?. Ecol. Indic..

[3] Almeida C., Mucha A.P., Vasconcelos M.T. (2011). Role of different salt marsh plants on metal retention in an urban estuary (Lima estuary, NW Portugal). Estuar. Coast Shelf Sci.

[4] Dias, S.M. (2018). Phytoremediation of Pharmaceuticals by Estuarine Salt Marsh Plants Universidade do Porto (Portugal).

[5] Paíga P., Santos L.H., Amorim C.G., Araújo A.N., Montenegro M.C.B., Pena A., Delerue-Matos C. (2013). Pilot monitoring study of ibuprofen in surface waters of north of Portugal. Environ. Sci. Pollut. Res..

[6] Ramos S., Ré P., Bordalo A.A. (2010). Recruitment of flatfish species to an estuarine nursery habitat (Lima estuary, NW Iberian Peninsula). J. Sea Res..

[7] Cunha J., Cabecinha E., Villasante S., Gonçalves J.A., Balbi S., Elliott M., Ramos S. (2024). Quantifying the role of saltmarsh as a vulnerable carbon sink: a case study from Northern Portugal. Sci. Total Environ..

[8] Ribeiro C., Couto C., Ribeiro A.R., Maia A.S., Santos M., Tiritan M.E., Pinto E., Almeida A.A. (2018). Distribution and environmental assessment of trace elements contamination of water, sediments and flora from Douro River estuary, Portugal. Sci. Total Environ..

[9] Rodrigues S.M., Silva D., Cunha J., Pereira R., Freitas V., Ramos S. (2022). Environmental influences, particularly river flow alteration, on larval fish assemblages in the Douro Estuary, Portugal. Reg. Stud. Mar. Sci..

[10] Azevedo I.C., Bordalo A.A., Duarte P.M. (2010). Influence of river discharge patterns on the hydrodynamics and potential contaminant dispersion in the Douro estuary (Portugal). Water Res..

[11] Madureira T.V., Barreiro J.C., Rocha M.J., Rocha E., Cass Q.B., Tiritan M.E. (2010). Spatiotemporal distribution of pharmaceuticals in the Douro River estuary (Portugal). Sci. Total Environ..

[12] Iglesias I., Almeida C.M.R., Teixeira C., Mucha A.P., Magalhães A., Bio A., Bastos L. (2020). Linking contaminant distribution to hydrodynamic patterns in an urban estuary: tthe Douro estuary test case. Sci. Total Environ..

[13] Rocha A.C.S., Teixeira C., Almeida C.M.R., Basto M.C.P., Reis-Henriques M.A., Guimarães L., Ferreira M. (2021). Assessing contamination from maritime trade and transportation on iberian waters: iimpact on Platichthys flesus. Environ. Sustain. Indicat..

[14] Cabral H.N., Fonseca V.F., Gamito R., Gonçalves C.I., Costa J.L., Erzini K., Gonçalves J., Martins J., Leite L., Andrade J.P., Ramos S., Bordalo A., Amorim E., Neto J.M., Marques J.C., Rebelo J.E., Silva C., Castro N., Almeida P.R., Domingos I., Gordo L.S., Costa M.J. (2012). Ecological quality assessment of transitional waters based on fish assemblages in Portuguese estuaries: the Estuarine Fish Assessment Index (EFAI). Ecol. Indic..

[15] Reis P.A., Salgado M.A., Vasconcelos V. (2013). Seasonal variation of metal contamination in the barnacles pollicipes pollicipes in northwest coast of Portugal show clear correlation with levels in the surrounding water. Mar. Pollut. Bull..

[16] Grasshoff, K.; Ehrhardt, M.; Kremling, K. Methods of seawater analysis; Verlag Chemie: Weinheim, Germany, 1983; Volume Second revised and extended edition; p. 419.

[17] Jones M.N. (1984). Nitrate reduction by shaking with cadmium: aalternative to cadmium columns. Water Res..

[18] Parsons T.R., Maita Y., Lalli C.M. (1984).

[19] Vohra, D. (1966). Determination of photosynthetic pigments in sea-water. Monographs onocéanographie methodology; UNESCO, Ed.; UNESCO: Paris, France, 66.

[20] APHA (1992).

